# EPOCK study protocol: a mixed-methods research program evaluating cancer care coordination nursing occupations in France as a complex intervention

**DOI:** 10.1186/s12913-019-4307-7

**Published:** 2019-07-12

**Authors:** F. Colombani, M. Sibé, M. Kret, B. Quintard, A. Ravaud, F. Saillour-Glénisson

**Affiliations:** 1 Centre de Coordination en Cancérologie (3C), CHU de Bordeaux (Bordeaux University Hospital), Groupe hospitalier Saint-André, 1 rue Jean Burguet, F-33000 Bordeaux, France; 2 Economie et Management des Organisations de Santé (EMOS), INSERM, ISPED, Centre INSERM U1219-Bordeaux Population Health, F-33000 Bordeaux, France; 3 ISPED (Bordeaux School of Public Health), Centre INSERM U1219-Bordeaux Population Health, Université de Bordeaux, F-33000 Bordeaux, France; 4 Service d’Information médicale, Unité de soutien méthodologique à la recherche clinique (USMR), CHU de Bordeaux, Pôle de Santé publique, F-33000 Bordeaux, France; 5 Faculté de Psychologie, Laboratoire EA 4136 Handicap, Activité, Cognition, Santé, Université de Bordeaux, F-33000 Bordeaux, France; 6 Pôle de cancérologie, Service d’Oncologie Médicale, CHU de Bordeaux, F-33000 Bordeaux, France; 7 Pôle de santé publique, Service d’Information Médicale, Unité Méthodes d’Evaluation en Santé, CHU de Bordeaux, F-33000 Bordeaux, France

**Keywords:** Care coordination, Continuity of patient care, Patient care management, Patient-centered care, Theoretical framework, Models, Nurses, Neoplasms, Organisational context

## Abstract

**Background:**

Facing the increasing cancer incidence and cancer survivorship, many national strategic cancer plans have identified cancer care coordination as a priority for health service improvement. However, the high variability of practices, the diversity of definitions and underlying concepts increases the existing difficulty to standardise, replicate, transpose and assess care coordination within the French health system context. The EPOCK national study aims at evaluating practices and the working context of hospital-based cancer care coordination nurses, based on a previously designed reference framework for care coordination within the French health system context.

**Methods:**

EPOCK is based on a comprehensive evaluation of nursing professions in cancer care coordination, considered as a complex intervention. Phase 1 (theoretical phase) will define and design a theoretical reference framework for care coordination in France through an international literature review, aiming to identify relevant models and all components of the expected framework and a structured consensus method, the Nominal group technique, aiming to select and prioritise the most relevant components already found in the literature review with regard to the French healthcare system; phase 2 (Operational phase) will consist in an in-depth analysis of practices, contexts, perceptions and attitudes related to care coordination occupations by nurses in oncology and all stakeholders (related professionals, patients and their caregivers) through a multicentric cross-sectional mixed-method evaluative study. The observed practices and contexts will be finally compared with the theoretical reference framework using both inductive and deductive approaches.

**Discussion:**

This study will result in an evaluation framework identifying key models and key elements relative to cancer care coordination interventions that can be used to guide management of cancer care coordination nursing occupations within the French healthcare system. EPOCK would also assist in public decision-making to identify optimal targets, skills profiles and scope of actions for cancer coordination professions. Finally, EPOCK will describe typology of nurse practices in cancer care coordination and thus obtain precise preliminary information essential for drafting a medico-economic evaluation study of these new nursing professions’ impact.

**Trial registration:**

Clinicaltrial.gov registration: NCT03350776, 11/22/2017.

## Background

### Care coordination has become a world-wide public health priority

Facing the growing ageing population and the increase in the number of people diagnosed with chronic conditions, coordination of care has become a crucial condition for care quality and safety [1–4]. Without good coordination of care, many patients, caregivers and families experience fragmented, poorly integrated care from multiple providers, often with suboptimal outcomes, inadequate sharing of clinical information, poor reconciliation of medicines, duplication of investigations and avoidable hospital admissions or readmissions [5–8] resulting in an increase in healthcare costs. Therefore, care coordination has been recognised as a key element of high-quality health care delivery and has become a global priority area for improving patient healthcare from prevention through disease management and complex case management [6, 9].

### Coordinating challenges in patients suffering from cancer’s care

Thanks to the progresses made in both diagnostic methods and therapy, the number of people living with cancer has dramatically increased over the past two decades [10–12]. Many cancer survivors end up with long term disabilities requiring ongoing care and support [13]. For many people, cancer survival now means living with a chronic and complex condition [14]. People with cancer are particularly at risk of receiving poorly organised and fragmented care due to the complex nature of the disease and its management, which often involves multidisciplinary care from a large team of medical, nursing and allied health practitioners in both hospital and community settings over extended periods of time. Care among multiple providers must be coordinated to avoid wasteful duplication of diagnostic testing, perilous polypharmacy, and confusion about conflicting care plans. Moreover, lack of care coordination is also associated with poor symptom control, medical errors, and high costs [15, 16]. Facing the increase of cancer incidence [17] and cancer survivorship, many national strategic cancer plans have identified the improvement of cancer care coordination as a priority for health service improvement [18]. However, attempts to improve care coordination are hindered by a lack of clarity about what “care coordination” actually encompasses [19].

### Care coordination remains a poorly defined complex concept

During the last decade different models of coordinated care have been widely applied and documented across a variety of settings, resulting in a multiplicity of care coordination definitions and conceptual frameworks [18].

#### Definitions of care coordination and related terms

Although the term “care coordination” is frequently used in the health services research literature, it is rarely clearly defined [20]. As a consequence, terms such care coordination, integration of care, transitional care, patient navigation, patient handoff, continuity of care, and patient-centered care are often used interchangeably [19, 21]. A literature review conducted by Armitage et al. uncovered some 175 overlapping definitions and concepts of care coordination, indicating the absence of consensus in its definition [22–24]. Furthermore, the Agency for Healthcare Research and Quality (AHRQ) identified more than 40 definitions of care coordination and concluded with a working definition of care coordination: *“Care coordination is the deliberate organization of patient care activities between two or more participants (including the patient) involved in a patient’s care to facilitate the appropriate delivery of health care services. Organizing care involves the marshalling of personnel and other resources needed to carry out all required patient care activities and is often managed by the exchange of information among participants responsible for different aspects of care.”* [23].

#### Care coordination models

Many healthcare systems facing with demographic, epidemiologic and organisational transitions have developed conceptual or operational care coordination models since the 80’s. These models provide several types of interventions ranging from individual Case management [25], through Disease management population-based programs [28], to fully Integrated systems (*i.e* the Chronic Care Model) [26–28]. The common approach by all these models is based on the case management program that is tailored to individual and focused on a single patient-centered care needs. Case management is schematically based on the ‘Plan (plan a change)-Do (implement this change)-Study (study the results)-Act (adjust the plan)’ cycle [29]. This healthcare quality improvement cycle therefore consists in several stages for each patient case [30]: Identifying a complex situation, assessment of the situation, consultation between professionals (multi-disciplinary or even multi-professional), and with the patient to take into account his priorities care planning interventions, identification of stakeholders and implementation interventions, monitoring of the effectiveness of interventions and reassessment of the situation. The level of management of each case is adapted according to the complexity of the patient, the condition of the person, the duration and intensity of the need for support [31]. The transition to a more integrated systems (concept proposed by Leutz) [32] is based on organisations networking or even “full integration” (management by a single entity and standardisation of rules and practices) [33] up to the Chronic Care Model [26, 34] which proposes a model for an entire health system. In these systems, patients are better informed and more mobilised in order to promote their “empowerment”. In addition, cooperation between health and social care organisations is thus well-recognised.

The latest emerged theoretical models entitled “clinical pathway” or “care pathway” [35] have been developed for people in chronic situations. These new models also take into account the interventions carried out in the person’s living environment. These models attempt to structure and standardise care processes [36].

#### Cancer care coordination models

Across the continuum of cancer care, patient navigation based on the Case management model (generally by a trained community member or a nurse case management) is the most frequent care coordination intervention worldwide [18]. It was initially developed to address structural barriers to continuous care (financial barriers, and transportation) [37]. This model has grown to patient navigator community-based programs and nationwide programs to address the psychological, social, and physical support systems that are mainly directed at improving the quality of life of patients with cancer [38, 39]. The transition to more integrated systems has required a shift from closer coordination of care for individuals to the formation of managed care organisations [32].

The most common new care-delivery model is the patient-centered medical home (PCMH), which has been developed by the AHRQ. This medical home model encompasses five components: comprehensive care, patient-centered care, coordinated care, accessible services, quality and safety. It is centered on enhanced care coordination to control the costs of care [40, 41]. At last, the Center for Medicare & Medicaid Innovation has developed the Oncology Care Model based on the Chronic care model [42], which aims to provide higher quality, more highly coordinated oncology care at the same or lower cost to Medicare. Oncology care model encourages participating practices to improve care and lower costs through an episode-based payment model that financially incentivises high-quality, coordinated care.

In the French context, the structuration of cancer care organisation has been implemented since 2000s through the National cancer plans. Cancer care quality assessments (announcement procedure, multidisciplinary, personal care plan, access to supportive care and coordination of care) are required in order to authorise French healthcare institutions to treat cancer patients. To ensure these national quality measures, several levels of cancer care coordination organisations have been implemented by The French Ministry of Health: 1) at a collective level (health services organisation), three structures have been created: the French National cancer Institute (the INCa), Regional oncology networks, and at a local level, Centres for cancer care coordination (3C); 2) at an individual level (patient care management), local health care providers have to facilitate continuity of care. Among them are general practitioners, local healthcare networks and new nursing positions. These nurses are called Cancer care coordination nurse (the French IDEC “*Infirmière Diplomée d’Etat de Coordination en cancérologie*”). They have been created to ensure continuity of care between hospital and patient’s home. Moreover, many other complementary approaches and nurse occupations have been introduced in France. They have been implemented empirically since 2000’s without underlying conceptualisation. The consequence of this non-concept based implementation is a large diversity of scope and function, reflected by the diversity of their job titles: NCCC (Nurse Cancer Care Coordinator focused on cancer complex case management), Pivot Nurses in Oncology (PNO) based on the Quebec model [43], IDEC (French cancer care coordination nurses) or IDECO (“*infirmier de coordination en chimiothérapie orale*”) specialised nurses supporting patients receiving oral chemotherapy, or AMA nurses for Ambulatory Medical Assistance in hematologic cancers-related patients based on patient navigation developed by Freeman in the United States [44].

This heterogeneity, particularly observed among nurses involved in hospital-to-city coordination, hampers their readability, understanding of their actions, definition of their mission and makes the evaluation of performance of cancer care coordination intervention very difficult.

Today, the high variability of practices, the lack of common definitions and underlying concepts increases the existing difficulty to standardise, replicate, transpose and assess cancer coordinated care [45, 46]. Furthermore, many authors acknowledge the lack of effective cancer care coordination and the need to standardise interventions [35, 47, 48].

### The EPOCK research program

Although the need to improve care coordination for people with cancer is widely recognised, efforts are hampered by the lack of common conceptual framework about care coordination and of information on the diversity of professional practices and contexts in care coordination [38]. Thus, we propose the Epock protocol consisting in comparing observed coordination of care practices, contexts and representations in France to a conceptual framework previously constructed. The project is focused on cancer care coordination nurses based at the hospital and linked to the city. This choice is justified because they are the most widespread, they have been introduced for a long time and have not yet been studied. In addition, in accordance with comments made by the jury experts of the PREPS call for projects, we had to focus on homogeneous practices in order not to scattered the evaluation.

The main objective of the Epock project is to evaluate practices and working context of hospital-based cancer care coordination French nurses, who are involved in hospital-to-city coordination, based on a previously designed reference framework for care coordination within the French health system context. The specific objectives are structured around three axes: [1] to develop a care coordination theoretical framework of healthcare coordination interventions in France that can be used to assist development, implementation, description, and evaluation of coordination care interventions in any clinical situation; [2] to describe practices, perceptions and job attitudes related to care coordination models in the context of oncology in France taking diversity and heterogeneity into consideration; [3] to compare the expected theoretical framework to professionals practices in order to propose a cancer care coordination measurement framework of cancer care coordination nurses’ intervention in France.

Throughout this paper, the term “care coordination” is used for “healthcare coordination” and refers to both health and social care. In addition, we consider the term “care coordination intervention” refers to coordination at the patient level (and not coordination at collective level) with reference to the World Health Organisation’s “people-centred care” definition [49].

This article presents the protocol of the Epock study.

## Methods/design

### Study design

**Fig. 1:**
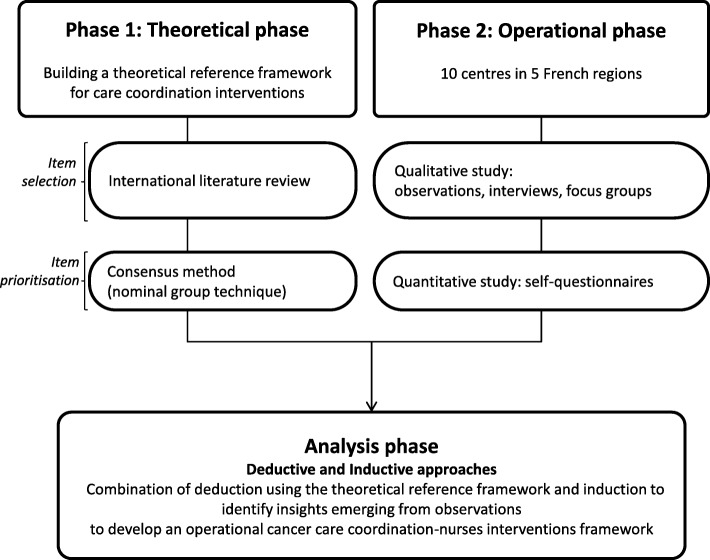
Epock study protocol flow chart, France

The Epock study is based on a comprehensive evaluation of nursing professions in cancer care coordination, considered as a complex intervention. This evaluation contains two phases (Fig. 1):

Phase 1 (Theoretical phase) consisting in defining and developing a theoretical standardised reference framework for care coordination in France, whatever the condition (care coordination for patients with multi-morbidity). This phase will be conducted in two steps: 1) an international literature review to identify relevant models and all the components of the expected theoretical reference framework, and 2) a consensus method using the Nominal Group Technique to select and prioritise the most relevant components already found in the literature review with regard to the French healthcare system. This preliminary phase will provide a standard for cancer care coordination evaluation; Phase 2 (Operational phase) consisting in analysing practices, contexts, perceptions (role tensions, work conditions, organisation support) and job attitudes (job implication) related to care coordination occupations by nurses in oncology through a multicentric cross-sectional mixed-method evaluative study (qualitative and quantitative analysis) [50]. This second phase will be carried out over a period of 36 months by collecting information from 10 French centres which have implemented various models of care coordination by nurses. The observed practices and context will be finally compared with the theoretical standardised reference framework using both inductive and deductive approaches.

#### Phase 1: developing a theoretical reference framework for care coordination interventions

##### Literature review

First, an international scoping literature review will be conducted about concepts and definitions of care coordination. The documents explored will cover agency reports and scientific articles in G*oogle, Google scholar, PubMed* and *Cairn*, proposing definitions of care coordination, concept analyses, models and frameworks of care coordination. Other documents will be selected by the snowball approach through our readings. We will search for English and French documents from 1990 to September 2018.

Then, we will conduct a systematic review of reviews of care coordination interventions effectiveness. The aim of this review is to provide descriptions of a wide variety of care coordination interventions. The research strategy will explore *Pubmed, Scopus and PsycInfo* databases using keywords focused on “care coordination” or “coordination of care” or “coordinated care”.

Included articles will be English and French language systematic reviews evaluating the impact of care coordination interventions, irrespective of clinical condition, patient population, or specific outcomes and published between 1990 and January 2018. Articles will be excluded if they did not describe care coordination interventions, if the elements of the coordination intervention and the criteria for evaluating this intervention have already been found. Furthermore, some articles could be added by snowballing approach (articles selected from the citations of the selected publications).

##### Nominal group technique

Finally, to select and prioritise the most relevant components of the reference framework of care coordination intervention, we will conduct a nominal group technique [51]. Experts will discuss and rate the relevance of each literature review retrieved component of the care coordination intervention. A component will be considered relevant if it contributes to care coordination in the context of the French health system. Expert group will represent various roles, missions, positions and implications in the coordination of care in France: researchers, regulators, practitioners, organisers and beneficiaries. Experts will be selected among: 1) Researchers who have published on care coordination, 2) Policy-makers and regulators that organise care coordination at local, regional and national levels, 3) Patients as experts [52] trained and graduated to support patients. The additional selection criteria for experts are volunteering, absence of conflicts of interest, geographic diversity. Between two to three experts in each category are expected to construct an expert group of ten to fifteen persons. Professionals of care coordination are excluded because they will be interviewed in the second phase of the EPOCK project (observational and descriptive phase of practices).

#### Phase 2: description of practices, contexts, perceptions and attitudes related to care coordination occupations by nurses in oncology (Table 2)

##### Population

The study population encompasses all nurses with a hospital-to-city coordination activity for cancer patients, EPOCK target nurses, and the professionals, patients and caregivers with whom they interact as part of their coordination activity. In order to capture the heterogeneity of the cancer coordination nursing professions and the diversity of contexts, we will consider all the categories of health care institutions, according to their status and size, in which these nurses have been implemented.

Study population combines the following different individuals and structures (Table 2). The identification of the different types of nursing professions leads us to select the centres where they have been implemented:

- **Target nurses**: Seven different subtypes of cancer care coordination nurses, with different designations, were identified and will be referred to as “target nurses” in the remainder of this paper. Target nurses designations were identified through publications in the literature (French or international papers published by French research teams) and by collecting operational feedback from French cancer professionals network experiencing cancer coordination nursing national program and expert opinion.
  - The “**IDEC**” (IDEC “*Infirmier Diplomé d’Etat de coordination en Cancérologie*”) are cancer coordination nurses who were first implemented in France.
  - The “**IDE DA/TAS**” nurses (“***I****nfirmier du* ***D****ispositif d’****A****nnonce ou* ***T****emps d’****A****ccompagnement* ***S****oignant*”) responsible for the support of delivering breaking bad news (nursing support for diagnosis announcement procedure) and for assessing supportive cancer care needs,
  - The “**IPO**” nurse (“***I****nfirmier* ***P****ivot en* ***O****ncologie*”) based on the Canadian model [43, 53, 54],
  - The “**AMA”** nurses (“***A****ssistance* ***M****alades* ***A****mbulatoires”)* for ambulatory patient support in hematologic cancers-related patients [42, 55, 56],
  - The “**COACH”** (“*infirmier* ***CO****ordination* ***A****ssistance* ***CH****imiothérapie*”) nurse telephone support at home during chemotherapy [46],
  - The “**IDECO”** (“***I****nfirmier spécialisés en* ***C****himiothérapie* ***O****rale”)* specialised nurses in oral chemotherapy,
  - The “**IDE HAD-CAD”** *(“****I****nfirmier coordonnateur d’****H****ospitalisation* ***A D****omicile pour les* ***C****himiothérapies* ***A D****omicile”)* coordinating nurse specialised in Hospital Care at Home for intravenous chemotherapy at home.
- **Centres.** We select all types of health care facilities existing in France: university or non-university centres, public or private, comprehensive cancer centres, and hospital care at home (HAD), in urban or rural areas, regardless of their size (large, medium and small centres). A total of ten centres were selected from five French regions because each of them has implemented a subtype of target cancer coordination nurse (Table 1). We will thus exclude: the PTAs (integrated platform for professionals facilitating patient’s orientation and professional’s coordination) that have been recently designed at a national level and implanted at a local level because they don’t coordinate from the hospital to the city, the healthcare and social service networks, private home-based care providers and healthcare houses.
- **Non-target cancer coordination nurses**. They will be identified by each centre in addition to the target nurses. They represent all the coordination nurses implemented in each selected centre. Won’t be involved other professions of cancer coordinators: coordinating medical doctors from care networks, coordinating medical doctors from hospital care at home, practitioners from centres for cancer care coordination (the French 3Cs), practitioners from French regional cancer networks. Nevertheless, these coordinators will be included as “referral professionals” to cancer coordination nurses (target and non-target).
- “**Referral professionals”** to cancer coordination nurses working inside the selected centre (hospital professionals: medical [oncologists, radiotherapists, surgeons]; paramedical [nurses, health officers], administrative [medical secretary, medical-administrative assistant], supportive oncological care [social worker, psychologist, dietician, physiotherapist ...], patient associations…) and outside (primary care providers and home care workers) who have activities related to cancer coordination nurses. They will be identified by target and non-target nurses.
- **Patients** with a pathway coordinated by a target nurse: adult patients with any types of cancer at any stages, who have been coordinated in the last month or have had at least 2 contacts with the cancer care coordinator nurse (patient-cancer care coordinator nurse pairs). Patients will be selected by target and non-target nurses already included in the Epock study (5 patients per coordination nurses).
- **Caregivers** chosen by patients already selected (patient-caregiver pairs): one caregiver per patient. If the patient is isolated, no relatives will be included.

**Table 1:** Epock project population: Selected target nurses and centres

N°	Target nurses	Target centres	Target territories	City	MSO size	Status of Health facilities
1	IDEC	University public hospital (extra-large size)	Region 1	Bordeaux	T5	Public
		Community public hospital (medium size)	Region 1	Libourne	T3	Public
		Private clinic (medium size)	Region 1	Bordeaux	T3	Private
2	IDEDA /TAS	Community public hospital (small size)	Region 1	Langon	T2	Public
		Private clinic (small size)	Region 2	Avignon	T1	Private
3	IPO	University public hospital (small size)	Region 3	St-Priest-En-Jarez	T1	Public
4	AMA	University public hospital (extra-large size)	Region 4	Toulouse	T5	Public
5	COACH	Comprehensive cancer centre (medium size)	Region 4	Toulouse	T2	private health institution with public interest
6	IDECO	Comprehensive cancer centre (medium size)	Region 5	Paris	T3	private health institution with public interest
7	IDE HAD CAD	Hospital care at home establishment (medium size)	Région 1	Talence	T3	private health institution with public interest
total	7 subtypes	10 centres	5 regions	8 cities	4 sizes	3 status

Region1: Nouvelle Aquitaine; region 2: Provence-Alpes-Côte d’Azur ; region 3: Auvergne-Rhône-Alpes ; region 4: Occitanie ; region 5: Ile-De-France

MSO size: Medicine, Surgery and Obstetrics size based on RSA (standardised discharge summaries) T1: [30 ; 6600 [ ; T2: RSA activity [6600 ; + 15000 [ ; T3: RSA activity [15000 ; + 40400 [ ; T4: RSA activity [40400 ; 90000 [ ; T5: RSA activity [90000 ; + infinity]

##### Study sample selection process

Within each centre, the target nurse will select:

- For the quantitative analyses:
  - 5 related professionals working inside the centre,
  - 5 related professionals working outside the centre,
  - 5 patients and their caregivers.
- For the qualitative analyses:
  - 10 related professionals working within the centre, including 5 professionals with regular contacts and 5 non-contact professionals (i.e. professionals with whom nurses should have contacts in the context of patient care but do not actually have any),
  - 10 related professionals working outside the centre (5 with regular contact ad 5 without any contact).

Within each centre, the non-target coordination nurses will select (for the quantitative study): 5 related professionals working inside the centre, 5 related professionals working outside the centre and 5 patients and their caregivers.

##### Study samples

Several samples will be constituted depending on the objective and type of study (qualitative and quantitative). We would expect 1148 observations divided into: 350 from qualitative study, 798 from quantitative study (Table 2):

- 10 on-site observations (3 days in each centre, i.e. 30 days on-site observations) of target nurses
- 2 focus groups
  - 1 “mono-professional” focus group with the ten target nurses (1 from each centre)
  - 1 “multi-stakeholders” focus group with a target nurse, related professionals (hospital and community-based), a patient and a caregiver
- 338 individual interviews
  - 38 cancer coordination nurses (target and non-target nurses)
  - 10 to 20 professionals linked to a target coordination nurse in each centre
  - 5 of the patients followed by a target coordination nurse in each centre
  - 5 caregivers per centre (1 caregiver per patient)
- 798 individuals interviewed by questionnaires
  - 38 cancer coordination nurses (target and non-target nurses)
  - 150 to 380 related professionals (5 to 10 related professionals per target and non-target nurses)
  - 190 patients (5 patients per cancer coordination nurses)
  - 190 caregivers (1 caregiver per patient)

**Table 2:** EPOCK project data collection: Study population, sample size, data collection tools, indicators collected, France

Study population	Sample size	Data collection tool	Analysis results and Indicators collected
*Qualitative study (18 months)*			
Target cancer coordination nurses	10 nurses observations (3 days per nurse, per centre)	Structured on-site observations: quiet observation of daily work, during 3 days with an Ad hoc observation guide	Relevant components of reference operational framework of cancer care coordination interventions for the French health care system: - Structure of the coordination intervention (organisational rules, human, financial, material, perceived resources, representations, beliefs) - Actors of coordination (role, competences …) - Process of care coordination and propositions of results - Environment, contexts of coordination
Target cancer coordination nurses	1 (1 day for the 10 nurses)	“Mono-professionals” target nurses focus group	
A cancer coordination nurse, a medical oncologist, a general practitioner, a home nurse, a patient and his caregiver	1 (1 day in 1 centre)	“Multi-stakeholders” focus group	
Cancer coordination nurses	38 (target cancer coordination nurses [n = 10] and all the others selected by the investigation centres [*n* = 28])	All providers’ semi-structured interviews	
Referral professionals to cancer coordination nurses	200 (20 professionals selected by the 10 target cancer coordination nurses)		
Patients	50 (5 patients selected by the 10 target cancer coordination nurses)		
Patients’ caregivers	50 (1 caregiver per patient)		
*Quantitative analysis* ad hoc *survey (18 months)*			
All cancer coordination nurses	38 (target and non-target cancer coordination nurses)	Self-administered questionnaires	Socio-demographic data, perceptions of organisational conditions
		Eisenberger Survey of Perceived Organizational Support (SPOS) score	Perception that organisation contributes to their work well-being by an adequate organisational support
		Rizzo’s questionnaire	Role perceptions (measures of role conflict and ambiguity)
		The workplace commitment Allen and Meyer’s scale	Measures of affective commitment, normative commitment, and continuance commitment
Referral professionals to cancer coordination nurses	380 (10 professionals per cancer coordination nurses)	Ad hoc self-administrated questionnaire	Satisfaction with coordination of care
Patients	190 (5 patients per cancer coordination nurses)	Ad hoc self-administrated questionnaire	Satisfaction with coordination of care
		EPICES Social precariousness score	Measure of deprivation
		Quality of Life Questionnaire – Core 30	Score of overall health status and quality of life; score reflecting levels of function domains and levels of symptom burden
Patients‘caregivers	190 (1 caregiver per patient)	Ad hoc self-administrated questionnaire	Satisfaction with coordination of care
		Zarit burden inventory	Measure subjective caregiver burden (estimating the total hours per week spent doing things for patients and how many hours caregivers missed paid work in the prior month due to caregiving responsibilities)

Sample estimations relative to the quantitative analysis were made from literature reference data to ensure sufficient accuracy for the main validated scales studied.

### Data collected (Table 2)

#### Qualitative study

##### Daily quiet structured observations of target nurses (1 on-site target nurse observation during 3 days per centre, i.e. 10 observations).

The target nurses will be followed by a research psychologist throughout their working day and all their activities will be recorded. This will describe the actual work of the various target cancer coordination nurses selected for the study, particularly in terms of activity, interlocutors, tools used and time spent on tasks. This will allow a first capture of the most accessible elements of their professions, to identify the first similarities or differences in their activities, and can be linked to other qualitative measures. These preliminary structured observations will be useful for adapting qualitative and quantitative collection tools. One target nurse per centre will be observed to get an overview of the full heterogeneity of practices (i.e. 10 cancer coordination nurses who represent their professions in the various centres).

##### Semi-structured interviews will be performed among cancer coordinator nurses, referral professionals (hospital professionals, community healthcare workers), patients and their caregivers.

Semi-structured interview guides will be built in part from the areas of the care coordination framework already identified in the implementation phase (phase 1). They will be performed to identify invariants and variants of practices of the cancer care coordination nurses, to capture the representations and feelings related to this coordination of cancer care, while taking into account the point of view of the different stakeholders involved in this coordination. We plan to include 10 target cancer coordination nurses and 28 other cancer coordination nurses (non-target) identified in the 10 centres, which represent the different types of cancer care coordination nurses from the centres studied. We also wish to interview 20 referral professionals (health professionals, medico-social and administrative professionals) who work in collaboration with these nurses: 10 hospital-based professionals (5 professionals who have interaction with cancer coordinating nurses, and 5 professionals with no interaction when they should have one), 10 city-based professionals (5 with and 5 without interaction with cancer coordination nurses), in order to have the most complete possible experience from these actors, which are involved in the coordination of care. Finally, we will include patients followed by coordination nurses and a caregiver (where possible), to gather the experiences of the primary beneficiaries of cancer care coordination. We set this number at 5 patients and caregivers (1 caregiver per patient) per centre, to ensure sufficient number of different opinions and capture as many varied representations as possible from patients and their relatives. A total of 338 individual will be interviewed. However, the investigating psychologists will take into account the saturation of the discourse (on the themes and perceptions) in order to possibly reduce the number of interviews or to reduce the duration of the interviews.

*Focus Groups* will provide additional and undeveloped information following interviews and questionnaires on the role of each cancer coordinating nurse. We will conduct two subtypes of focus groups: a) a multi stakeholder’s focus group including hospital-based professionals (a target cancer coordination nurse, an oncologist, a clinical care nurse) and city-based ones (a general practitioner, an independent nurse, a pharmacist) with a patient and a caregiver’s: after having collected qualitative information from each actor separately, we will dynamically highlight the perceptions of the different actors involved in or concerned by cancer care coordination. Focus groups could assess the points of convergence or divergences relating to the missions of the cancer coordination nurses and stakeholders needs; b) A “mono-professional” focus group between target coordination nurses: it will concern all the target professionals identified in each centre (1 target nurse per centre, *n* = 10) and will allow a direct comparison of the similarities and differences between these new coordination nursing professions, allowing participants to share their experiences with each other and to compare it.

Saturation of themes will be used as a criterion for discontinuing data collection (Table 2).

*Quantitative study* will provide a quantified description and analysis of the psychosocial characteristics of these new professions, both in terms of organisation, perception and job attitudes of cancer coordination nurses, quality of life of patients, reduction of the burden on caregivers, but also in terms of satisfaction with the coordination intervention by all stakeholders involved in the intervention. Quantitative data collection mostly relies on international standardised scales and Ad hoc questionnaires among all providers.

Eisenberger Survey of Perceived organizational support score [57] will be used to measure the cancer coordination nurses’ perception concerning the extent to which the organisation values their contribution and cares about their well-being. The Meyer and Allen’s model [58, 59] proposes that organisational commitment is experienced by the employee as three simultaneous mind-sets encompassing affective, normative, and continuance organisational commitment. Affective commitment reflects commitment based on emotional ties the employee develops with the organisation primarily via positive work experiences. Normative commitment reflects commitment based on perceived obligation towards the organisation, for example rooted in the norms of reciprocity. Continuance Commitment reflects commitment based on the perceived costs, both economic and social, of leaving the organisation.

Among professionals working with cancer coordination nurses (referral professional), we will measure satisfaction of care coordination with an Ad Hoc questionnaire due to the lack of validated scale in French language. This questionnaire will be developed from a satisfaction survey conducted by the French National Cancer Institute and the French Ministry of Health [60].

The Quality of Life Questionnaire Core 30 items (version 3.0) from The European Organisation for Research and Treatment of Cancer (EORTC) [61] will be used to measure patient’s perceptions of the impact of their illness and treatments on their well-being. This questionnaire (appropriate for self-administration, *i.e* brief and easy to complete) is composed of 5 multi-item function scales (physical, role, cognitive, emotional, and social) and 9 symptom scales (fatigue, pain, and nausea and vomiting) and a number of single items assessing additional symptoms commonly reported by cancer patients (dyspnoea, loss of appetite, insomnia, constipation and diarrhoea) and perceived financial impact of the disease. The questionnaire is validated in its French version and is specific to cancer.

The Zarit burden inventory will be used to self-assess the material and emotional burden felt by caregivers [62]. It has been reported in caregivers of cancer patients [63, 64].

### Data collection process

The quantitative and qualitative data collections will be performed at the same time across the ten investigation centres located in five French regions.

Structured observations, semi-structured interviews and focus groups will be conducted by a team of research psychologists in all the ten centres.

All questionnaires will be collected within each centre, according to the same procedures. A project manager will coordinate data collection with a collection’s referent within each centre. This referent will be the health executive or the person designated by the head of the department or the principal investigator from the centre in which the coordination professional is located.

#### Data collection from all cancer care coordination nurses

Target and non-target nurses will be contacted directly by the project team to complete their questionnaires (self-administrated questionnaires). Questionnaires will be distributed in a sealed envelope by investigators to all coordination nurses in each centre (target and non-target coordination nurses). These nurses will have 2 weeks to answer the questionnaires (with several reminders by phone or email every 15 days over 3 months). Questionnaires will be returned directly to the project team at the promoter centre. Questionnaires will be enclosed in a sealed envelope with a label containing an anonymous identification number previously established by the study’s project manager and the data manager. This professional’s identification number will not be known by the department’s health officer.

#### Data collection from professionals (hospital and city) related to coordination nurses

Cancer nurse coordinators (target and non-target) will identify their related professional (5 professional working inside the centre, and 5 outside). After obtaining their agreement, the Epock project manager will send self-questionnaires to these professionals with a prepaid reply envelope. They will have 3 weeks to answer. The Epock project manager will at last contact them by telephone to retrieve their questionnaires (two telephone reminders at two-week intervals, then a reminder at 2 months, and a final mailing at 3 month).

#### Data collection from patients and caregivers

Coordination nurses will have to select patients and their caregivers by handing them self-administrated questionnaires. Patients and their caregivers’ will complete self-questionnaires before or after the nursing time support with the coordinating nurse. Patients will return their questionnaires to nurses, and nurses to the promoter. Patients and caregivers could also return their questionnaires directly to the project team using a prepaid envelope.

In addition, for the 5 patients identified by coordination nurses in each centre, the coordination nurses will complete an anonymous questionnaire containing data on age, individual deprivation (with the French “Evaluation of Deprivation and Inequalities in Health Examination Centres” score-the EPICES score) [65], type of primary tumour (International statistical Classification of Diseases and Related Health Problems 10th Revision, in French language) [66], stage of the disease (with the Tumor-Node-Metastasis [TNM] classification of malignant tumours) [67] at the time of management by the coordination nurse. The coordination nurses will be responsible for following up with patients and caregivers to retrieve their questionnaires.

The EPOCK study protocol was approved by institutional review boards and Ethics Committees (Comité de Protection des Personnes [CPP] du Sud-Ouest et Outre-Mer III number: 2017-A02049–44).

The investigator or the coordination nurse targeted by centre offers to participate in this research to the participant / legal representative. He informs him of the purpose, the computerised processing of his data will be collected during this research and also specifies his rights of access, opposition and rectification to these data. Information notes will be given to patients, their relatives and the healthcare professionals requested. The investigator or coordinating nurse will also check the eligibility criteria. If the person agrees to participate, he or she gives his or her consent orally. In case of participation agreement is collected from the representative, the participant will be informed as soon as possible and asked for his or her participation agreement for the possible continuation of this research and for the use of the data concerning him or her that are collected in the context of this research. The participant may, at any time, object to the use of his or her data for research purposes.

### Analysis

Qualitative data will be recorded, anonymous, integrally transcribed and imported into NVivo12 software to achieve thematic and semantic content analyses, using analyses of similarity and correspondence factorial analyses. Two analytic team members will independently code the initial interview for each participant interviewed to clarify meanings of codes and come to consensus when disagreements occur, thus defining the initial code book [40]. The construction process of these thematic categories, coding, is both inductive and deductive because the development of themes and sub themes rests on both literature and emerging categories of empirical analysis. Qualitative analysis could draw a typology of the various cancer care coordination professions’ representations.

Quantitative data will consist in describing contextual factors as organisational contexts, clinical contexts, social contexts … and analysing associations between contextual factors and either satisfaction regards to care coordination for patients, caregivers and referral professionals, or quality of life at work (for cancer care coordinator nurses). Quantitative variables will be described in terms of numbers, percentages, and 95% confidence intervals according to the exact binomial distribution. Comparisons will be made by the Chi-square, corrected Chi-square or exact Fisher test. A logistic or polytomial regression model is used to take into account the adjustment variables. The quantitative variables will be described in terms of mean, standard deviation, median, extent and interquartile range. Comparisons will be made by Student test, Student test for unequal variances, Wilcoxon test, according to the distribution of the variable of interest.

Mixed-methods analysis: The both qualitative and quantitative analyses will be performed simultaneously over the same period of time (two concomitant analyses). Then, some results obtained in the qualitative analysis could be quantified and introduced into the final quantitative analysis. At last, qualitative and quantitative methods will be combined in a convergent interactive analytic design (data triangulation) to examine interdependencies between reference framework and observed elements. All data will be analysed according to the theoretical reference framework with the ability to allow new themes emerging from the data to be identified. This approach is a combination of deduction using the theoretical framework and induction to identify insights emerging from the data. The development of this new operational model will result in a comparative analysis between observed practices and a theoretical model.

### Monitoring and coordination

The Steering and scientific committees of EPOCK include oncologists, epidemiologists, researchers on management and organisation sciences and health psychology.

The EPOCK’s interdisciplinary research team was assembled because of its experience and expertise in evaluating complex interventions (expertise in qualitative and quantitative research methods, appropriate interdisciplinary theoretical expertise) [50].

The Epock study is ongoing. Investigators are still collecting data.

## Discussion

### Expected results

This paper presents a new protocol aiming at evaluating cancer care coordination interventions through a new model by comparing a theoretical reference framework of care coordination with practices in oncology within the French Healthcare system.

The Epock project aims to address the lack of information on coordination of care in France by three main outputs:

1. Building a theoretical reference framework for care coordination in France

The EPOCK Phase 1 will identify key elements contained in a reference framework for care coordination interventions in any clinical situation (multiple chronic conditions, physical or mental and long-term care patients). This reference framework will help the Epock research group to build in part the qualitative study tools needed in the second step of the Epock study (observation and interviews guides), and to analyse the practices of care coordination nurses in the specific context of oncology in France. It could also be useful in other clinical contexts and could help to compare care coordination programs in various contexts; to develop position profiles and job description for care coordination workers and to develop criteria for evaluating best practices among care coordination professionals and work environment. It could be used to build a wide range of teaching materials and training programs from under-graduate to post-graduate programs and lifelong learning courses. It could also be used by decision makers in order to develop care coordination evidence-based clinical indicators.

2) Analysing in fine details practices and contexts of the reality of nursing coordination professions in France

This mixed-methods analyses will allow us to define relevant components of cancer care coordination interventions within the context of the French health care system: a) Structural dimension of the coordination intervention (organisational rules, human, financial, material, symbolic resources, representations, beliefs); b) Actors of coordination: roles, competences, their interactions with the other healthcare stakeholders, their qualifications (competence grid on knowledge, know-how); c) Process of care coordination: collection of the nature of the coordination tasks associated with the workstation (types of care, types of practices, types of activities) and their purposes (purpose and objective of coordination), with the possibility of modelling the process and the expected results of this coordination; d) Environment and context of coordination: clinical, organisational and managerial contexts with which these coordination professions interact.

The immediate expected outcome of the Epock study will be to provide information to define roles and duties, requirements and optimal care contexts of the coordination nursing professions and thus could contribute to harmonising practices for this profession.

3) Designing an operational model consisting of recommendations that will help to align the profession of cancer care nurse-coordinator with the theoretical reference framework

This study will result in an evaluation framework identifying key model elements, barriers, and facilitators that can be used to guide cancer care coordination interventions in the French healthcare system in the absence of a systematic and uniform referral system for cancer care coordination intervention. The EPOCK study will further our knowledge of the process of cancer care coordination intervention. This could allow us to accurately define the elements of nurse intervention processes and their alignment with the theoretical model (what nurses do, what they should do …) and thus draw up an operational model for the coordination of cancer care in France that could be useful in many purposes:

- Contribution to practices in oncology: it would help to describe and implement programs of cancer care coordination intervention, to harmonise practices (with job profile description for cancer coordination nurses), to assist public decision-makers to identify the optimal targets, profiles and scope of action for cancer coordination professions
- Implications for teaching and education: there is currently an increasing number of training courses in France on the coordination of cancer care, but none are theory-based. It would be appropriate to develop a dedicated university degree for cancer coordination nurses based on the reference framework and the nursing profession profile found in the Epock project.
- Implications for the management and organisation of cancer care: comparing programs for cancer care coordination by developing evaluation criteria of care coordination nurses best practices; for decision makers in order to develop cancer care coordination evidence-based clinical indicators
- Implications for research: it should produce key elements for cancer care coordination intervention effective implementations and for designing further medico-economic evaluation of cancer care coordination intervention impact

### Strengths and limitations

Process evaluation of complex interventions usually requires a combination of quantitative and qualitative methods in order to provide a broader perspective. There is a strong need for a theoretical model of care coordination to provide a reference framework against which to observe cancer care coordination practices in France. This theoretical model will help the Epock research team to determine care coordination framework dimensions that will be later appropriate for scaled survey questions (quantitative analysis) and interview questions (qualitative analysis). In order to build this theoretical model we will use an inductive process conducted iteratively using a review and a structured consensus method. The international systematic review on the impact of care coordination interventions will be carried out to fully describe all dimensions and detailed elements which can be included in this reference framework. The search will be as wide as possible to collect a very large and comprehensive description of care coordination interventions related to the contexts, activities, actors, tools and impact of this complex intervention. As the term “care coordination” is not part of the MeSH (Medical Subject Headings) terms, care coordination is often approached by combinations of other terms such as “comprehensive care”, “case management”, “care integration” or “continuity of care”, “coordinated care” or “integrated care”. However, these terms are not specific to care coordination and will not answer our research question. In addition, these terms further confuse the definition of care coordination. To avoid making any a priori assumptions about the definition of care coordination, we have chosen, for the research equation, to include only terms that specifically contain the word “coordination” or “coordinated”. The review team will include at least one person with methodological expertise in conducting systematic reviews and one expert of knowledge. We will limit the search to the period from the early 1990s onwards and this will enable us to identify all but the very small minority of systematic reviews conducted before then [46].

We choose a nominal group technique to provide group dynamics by direct interactions between experts, and structure a consensus by giving equal weight to each participant. The nominal group technique accompanied by multi-voting technique is a well-established, structured, multistep, facilitated, group meeting technique used to generate a consensus and prioritise relevant elements [41, 47]. We will ensure that all stakeholders are represented, including patients as experts [52].

Qualitative studies are methods promoted in the field of complex interventions to refine analysis of “care coordination processes”. In general, the choice of a qualitative method is appropriate for a comprehensive perspective of a given phenomenon, which is, here, to describe and analyse the “why” and “how” of cancer care coordination, in its ‘natural’ (in field contexts) context. The coordination of cancer care is sufficiently innovative or not yet stabilised to justify its use. The qualitative study design follows the COREQ checklist [68]. Objectivity of qualitative methods is based on triangulation of information sources (observations, interviews and focus groups). Triangulating such data strengthens validity and will help to analyse all stakeholders’ representations, perceptions and satisfaction with cancer care coordination. These data will present an in-depth examination of the role of health professionals in coordinating cancer care, their related professionals and also patients and caregivers’ priorities and preferences. This qualitative approach will be complemented by a quantitative analysis.

Quantitative methods (by questionnaires) are appropriate insofar as they seek to quantify, explain and predict a phenomenon that is sufficiently known and for which content to be measured is relatively stable or standardised. Results of these standardised scales should highlight the main organisational characteristics of the coordination nursing professions and link them to their attitudinal effects, taking into account the main psychosocial risks currently associated with the quality of life at work and the effectiveness of nurses’ work. These measures are an important component to the study, which aims to understand and describe these new professions in their entirety. All the selected scales are validated and used regardless of organisational or cultural contexts. The majority of them have been used previously by researchers in French healthcare system and have been validated in French.

For professionals working with coordination professionals, as well as the patients and their caregivers that we will follow, we plan to add a measure of coordination satisfaction and management by a short questionnaire designed ad hoc, in the absence of an established measurement scale.

The added value of the quantitative approach is primarily to capture the psychosocial perceptions of coordination professionals regarding their work environment and quality of life at work. We hypothesise that perceived organisational support and role tensions (conflict and ambiguity) could influence their level of performance and involvement in their role. These quantifiable elements, which have already been studied in the managerial and psychosocial literature in other contexts [69], contribute to the exploration of coordination nurse professions. Despite the heterogeneity of coordination practices, these results will highlight the psychosocial effects induced by these new professions and will make it possible to identify levers for organisational and managerial action.

Quantitative analyses will be complemented by qualitative analyses in order to identify potential factors associated with the quality of life at work or patients’ quality of life. Quantitative analyses will explore the relationships between the different variables measured: relations between perception of the work context (organisational support, role tension), sense of personal effectiveness, quality of work life and patients’ quality of life. Finally, principal component analysis and hierarchical clustering analysis will be conducted to identify typologies based on pre-analyses items already found in both qualitative and quantitative analysis.

The process of theorising is enhanced when qualitative and quantitative methods are mixed and inductive and deductive processes are combined [70, 71]. During Epock phase 1 (inductive methods), application of inductive methods will involve reviewing the empirical data that emerged from the overview of reviews. Variables identified in the consensus group will define care coordination intervention reference framework. During Epock phase 2 (deductive and inductive methods), we will use a combination of deduction using the theoretical framework already found in phase 1 and induction to identify insights emerging from the observed data [72]. Results from phase 2 will list components of the operational cancer care coordination model and their links with the theoretical framework [73]. This could lead to recommendations on the content of the model (process, job profile, skills …). Mixed-methods provide a high potential for such a modellisation [74].

Mixed-method strategies can offset both inductive and deductive analysis weaknesses by allowing for both exploration and analysis in the same study. Combining methodologies helps to reduce the personal biases of the researcher.

### Justification of study population

In order to capture the heterogeneity of the cancer coordination nursing professions and the diversity of contexts, we chose a national multicentre study targeting all categories of health care institutions (according to their status and size) in which these professions have been set up. The 10 centres included in Epock study are geographically spread in five French regions on the whole French territory and have both rural and urban areas. They represent the geographical diversity of the French territory practices. The choice to focus the study solely in health facilities and nurse functions is justified by the concern to make the measure more reliable by referring to relatively identical work contexts (health care institutions) and professions with a common set of skills (nurses). In addition, nurses are the main workforce in the cancer care coordination professions in France and they represent a new profession to be evaluated. One of the limitations of our study is that we do not have the whole diversity of nursing occupations in each selected health care facility. However, it was important to reduce the scope of the project in terms of feasibility with regard to the sample size (1200 cases observed) and timing of the project (36 months planned).

### Feasibility for project implementation

Modelling the concept of care coordination requires crossing perspectives to accurately analyse this complex intervention [49, 50]. Our multidisciplinary research team combines all the competencies required for the EPOCK study with the expertise in:

- human and social sciences with researchers in psychology, management sciences
- epidemiology with medical methodologists in health evaluation, experts in health services research, biostatisticians
- cancer field domains: medical oncologists, nurses, medical coordinators from cancer coordination centres

The Health Evaluation Methods Unit (UMES) and the Research Methodology Support Unit (USMR) of the Bordeaux University Hospital, the Laboratory of Psychology of Bordeaux (EA 4136: Handicap, Activity Cognition, Health) and the Economic and Management of Healthcare organisations (EMOS) research team of the U1219 INSERM-University of Bordeaux Centre (Bordeaux Population Health) are responsible for the overall coordination of the project with the Cancer Coordination Centre (3C) at the Bordeaux University Hospital.

The multidisciplinary Epock research team has a strong experience in the management of such a project and is integrated into a network of actors in oncology in France which will facilitate the constitution of the nominal group and the voluntary work of the centres.

In addition, the feasibility of the study will be guaranteed by the collection of all the participation agreements of the investigator centres (via heads of departments that have set up the cancer coordination nurses in their centre), and a planned project monitoring governance. Research monitoring will be carried out by a steering committee (2 per year), a scientific committee (1 per month) and bi-weekly meetings of the operational team.

Epock is a challenging French national research project for the development of an evidence-based reference framework for cancer care coordination interventions considered as a complex intervention. The description of this complex intervention has many methodological challenges: develop a common evaluation framework; clarify how it was used on-the-ground; disseminate findings to policy and practice stakeholders.

We will be able to support health care management and policy decision making in order to implement cancer care coordination nurses at health facilities level. We will also be able to produce an evaluation for cancer care coordination interventions.

The next step will be to conduct a medico-economic evaluation of the impact of these new care coordination nursing professions thus defined.

## Data Availability

Not applicable.

## Abbreviations

3C: Centre de Coordination en Cancérologie
AHRQ: Agency for Healthcare Research and Quality
AMA nurse: Ambulatory Medical Assistance nurse
CAD: Chimiothérapie A Domicile
COACH: Infirmier de COordination Assistance en CHimiothérapie
EORTC: European Organisation for Research and Treatment of Cancer
EPICES: Evaluation de la Précarité et des Inégalités de santé dans les Centres d’Examens de Santé
EPOCK: Etude Pilote Observationnelle et analytique des pratiques et organisations associées aux nouveaux métiers de la Coordination des soins en cancérologie (K)
HAD: Hospitalization A Domicile
ICD-10: International Classification of Diseases 10th Revision
IDE: Infirmier Diplômé d’Etat
IDEC: Infirmier de Coordination en cancérologie
IDECO: Infirmier spécialisé en Chimiothérapie Orale
INCa: Institut National du Cancer
MSO: Medicine, Surgery, and Obstetrics facilities
NCC: Nurse Cancer care Coordinator
PCMH: Patient-Centered Medical Home
PNO: Pivot Nurse in Oncology
PREPS: Programme de REcherche sur la Performance du Système des soins
TNM: Tumor Nodes Metastasis Classification of Malignant Tumors
UICC: Union for International Cancer Control
WHO: World Health Organisation
